# The effect of improved hand hygiene on nosocomial MRSA control

**DOI:** 10.1186/2047-2994-3-34

**Published:** 2014-11-26

**Authors:** Kalisvar Marimuthu, Didier Pittet, Stephan Harbarth

**Affiliations:** Institute of Infectious Diseases and Epidemiology, Tan Tock Seng Hospital, Singapore, Singapore; Infection Control Program, Geneva University Hospitals and Faculty of Medicine, 1211 Geneva 14, Switzerland; Infection Control Program and WHO Collaborating Center on Patient Safety, Geneva University Hospitals and Faculty of Medicine, Geneva, Switzerland

**Keywords:** Hand hygiene, Hand washing, Multimodal strategy, Alcohol-based handrubs, Nosocomial MRSA, MRSA control, MRSA bacteremia

## Abstract

The purpose of this review is to examine studies that have assessed the association between hand hygiene enhancement and methicillin-resistant *Staphylococcus aureus* (MRSA) rates and to explore controversies surrounding this association. Many studies have been published confirming the link between improved hand hygiene compliance and reduction in MRSA acquisition and infections, including bacteremia. These studies have also shown the cost-beneficial nature of these programmes. Despite considerable research some issues remain unanswered still, including the temporal relationship between hand hygiene enhancement strategies and decrease in MRSA rates, association between hand hygiene enhancement and MRSA-related surgical site infections, diminishing effect of hand hygiene compliance on MRSA rates after reaching a threshold and the role of instituting contact precautions in the setting of low MRSA rates and sufficient hand hygiene compliance. In conclusion, enhancement of hand hygiene compliance has been shown to reduce MRSA rates; however, some open issues warrant further investigation.

## Introduction

**Figure 1:**
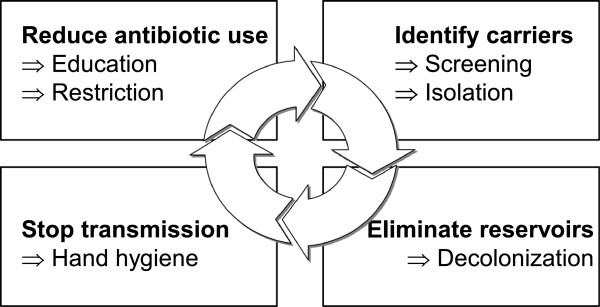
**Strategies to control nosocomial methicillin-resistant** ***S. aureus.*** Adapted with permission from Harbarth [1].

It has been more than a century since Ignaz Semmelweis’s discovery that healthcare workers’ hands could potentially transmit infections to patients. Semmelweis’s uncelebrated death in an asylum was vindicated by a slew of evidence that emerged later, and continues to emerge until now, showing clear association between hand hygiene and healthcare associated infections (HAI), especially those related to MRSA (Table 1). Clonal spread of MRSA is facilitated by cross-transmission via the hands of healthcare workers and exacerbated by the selection pressure exerted by broad spectrum antibiotic treatments [1]. Consequently, control of endemic MRSA generally revolves around reduction of antibiotic usage, screening and contact isolation of MRSA carriers, decolonization and improvement of hand hygiene compliance (Figure 1). While opinions differ with regard to the best infection control method, hand hygiene is considered the cornerstone by many experts [2]. This review focuses on summarising existing evidence on the role of hand hygiene on MRSA control.

**Table 1:** **Selected studies that specifically assessed the role of hand hygiene enhancement on methicillin-resistant** ***Staphylococcus aureus*** **rates**

First author and year	Trial design	Setting	Hand hygiene enhancement strategy	Other interventions to reduce MRSA
Pittet et al. [3]	Quasi-experimental	Hospital-wide	ABHR, staff education, reminders, performance feedback and administrative involvement	On-site surveillance, implementation of prevention guidelines, outbreak investigations, and environmental sanitization
Johnson et al. [5]	Quasi-experimental	Hospital-wide	ABHR, staff education, reminders, performance and feedback and culture change program	Enhanced cleaning of healthcare equipment, and decolonization of MRSA patients
Grayson et al. [7]	Quasi-experimental	Multiple hospitals	ABHR, education, performance feedback and recommendations for culture change	Individual hospitals observed various MRSA control measures
Stone et al. [10]	Prospective ecological	Acute NHS hospital trusts, United Kingdom	ABHR, reminders, audit and performance feedback and patient empowerment	Saving lives campaign, Health Act 2006, and visit to trusts by Department of Health improvement team
Kirkland et al. [11]	Before and after study	Hospital-wide	Leadership accountability, measurement/performance feedback, ABHR, education/training and marketing/communication	None reported
Lee et al. [15]	Prospective interventional cohort study	Surgical wards	Hand hygiene improvement program as per WHO guideline	Screening and contact isolation and targeted decolonization
Derde et al. [17]	Hybrid prospective interventional cohort study and RCT	Intensive care units	Hand hygiene improvement program as per WHO guideline	Universal decolonization in phase 2 and screening and isolation in phase 3

*Abbreviations: MRSA* methicillin-resistant Staphylococcus aureus, *ABHR* alcohol-based handrubs, *NHS* National Health Service, *RCT* randomized controlled trial.

## Review

### The evidence and the known

The ultimate aim of MRSA control strategies is to prevent MRSA clinical infections, especially MRSA bacteremia. In 2000, Pittet and colleagues from University of Geneva Hospitals, Switzerland conducted a quasi-experimental interventional cohort study to assess the effect of enhancement of hand hygiene compliance on MRSA transmission and nosocomial infection rates [3]. A combination of visual reminders, increasing access to alcohol-based handrubs (ABHR), hand hygiene performance monitoring and feedback to hospital staff and senior management support resulted in an increase of hand hygiene compliance from 48% in 1994 to 66% in 1997. During the same period, incidence of MRSA bacteremia and MRSA clinical cultures decreased from 0.74 to 0.24 episodes per 10000 patient-days (p < 0.001) and 2.16 to 0.93 episodes per 10000 patient-days (P < 0.001), respectively. Investigators also observed a significant year-on-year reduction in MRSA acquisition (p = 0.021). Hand hygiene enhancement was implemented as part of a multimodal infection control strategy and the design of the study precluded apportioning of benefit to specific elements of the strategy. However, it is noteworthy that hand hygiene enhancement was the only incremental intervention introduced during the study period. Additionally, investigators estimated that an average of US$ 1.42 per patient admitted was required to support the MRSA prevention programme and concluded that the hand hygiene multimodal promotion strategy was cost-saving if even 1% of the reduction in HAI observed could be attributed to improved hand hygiene practices. In 2004, Pittet and colleagues evaluated the long-term costs associated with the hand hygiene promotion campaign and found that the campaign’s total costs corresponded to less than 1% of costs attributable to HAI [4].

The adaptability and effectiveness of the Geneva multimodal intervention model was reiterated by an Australian group in 2005 under advice of the University of Geneva infection control team [5]. The Australian investigators implemented an infection control bundle consisting of enhancement of hand hygiene compliance with ABHR, decolonization of MRSA carriers, enhanced cleaning of healthcare equipment and hospital-wide culture change program for 3 years to control endemic MRSA. During the first 12 months, the hand hygiene compliance rate improved significantly from 21% to 42% (p < 0.001). Interestingly, in the first 28 months MRSA clinical isolates and MRSA bacteremia remained static. Moreover, the MRSA colonization rate did not change at 12 months post-intervention. By 36 months post-intervention, however, the MRSA clinical isolates per 100 patient-discharges per month declined by 40% (95% CI, 23% - 58%; p < 0.001) while the patient-episodes of MRSA bacteremia declined significantly (p = 0.003) compared to pre-intervention period. Subsequently, the group implemented a centrally coordinated, multisite hand hygiene culture-change program in Victorian healthcare institutions and assessed its effect on MRSA bacteremia [6]. Encouragingly, with increase in hand hygiene compliance by about 30%, the incidence of MRSA bacteremia reduced from 0.03 per 100 patient-days per month to 0.01 per 100 patient-days per month (p = 0.09 for trend) at 12 months. It is important to note that the ABHR used both in the original “Geneva multimodal programme” as well as in the Australian interventions contained chlorhexidine (0.5%).

Riding on these impressive results, the Australian National Hand Hygiene initiative (NHHI), also known as “Hand Hygiene Australia”, was launched in 2009 and two years later investigators documented a significant decline in national MRSA bacteremia rates (p = 0.008). Although the changes in total MRSA bacteremia rate during 2009–2010 cannot be definitively linked to NHHI, they are in line with previous Australian and international reports [7]. More recently, the impact of the NHHI on healthcare-associated *S. aureus* bacteremia was investigated by Barnett and colleague [8]. Four out of 6 states noted a reduction in infection rates. Varying degree of change in infection control measures resulted in different rates of response with 2 states showing immediate reduction and another 2 states showing linear decrease in infection rates. Two states, which already had an established initiative with low MRSA infection rates before the implementation of NHHI did not show further decline.

Investigators from the United Kingdom employed statistical models to investigate the hospital-level relationships among MRSA prevalence, antibiotics use and infection control policies and practices across Europe [9]. Adjusted linear regression analysis showed that lower MRSA prevalence was associated with use of ABHR for hand hygiene (mean difference 10.3%, 99% CI 1.2 – 10.3), and placement of MRSA patients in single rooms (mean difference 11.2%, 99% CI 1.4 – 20.9). However, after adjusting further for geographical variation, the single strongest predictor that remained was the use of ABHR. While response bias cannot be ruled out as the participating hospitals were self-selecting, the fact that this was a large study spanning the whole of Europe mitigates the risk and makes the study more generalizable.

In 2004, the CleanYourHands campaign was rolled out to healthcare workers in all acute National Health Service (NHS) hospital trusts in England and Wales to control the rate of MRSA, methicillin-sensitive *S. aureus* (MSSA), and *Clostridium difficile* infection [10]. The campaign had three predefined phases: 1 July 2004 to 31 December 2004 (before roll out), 1 January to 30 June 2005 (campaign roll out) and 1 July 2005 to 30 June 2008 (after roll out). An ecological study done to assess the effect of this campaign was published recently and revealed that as the campaign moved along the 3 phases, procurement of soap and ABHR tripled. Increased procurement of ABHR was independently associated with reduced MRSA bacteremia, but only in the last four quarters of the study (adjusted incidence rate ratio for 1mL increase per patient bed day 0.990, 95% CI, 0.985 to 0.995; p < 0.0001). However, increasing procurement was not the sole driver of falling MRSA bacteremia as publication of Health Act 2006 and Department of Health improvement team visits, which happened at the same time, were both strongly correlated with falling MRSA rates.

A 3-year, multifaceted, sequential implementation of hand hygiene enhancement intervention at a US teaching hospital resulted in an increase in hand hygiene compliance from 41% to 87% (p < 0.01). This was accompanied by a significant and sustained reduction in healthcare-associated *S. aureus* bacteremia from 2.1 to 1.4 per 1000 patient-days (p = 0.004). Contrary to expectations, *S. aureus* infections attributable to the operating room which were expected to be less sensitive to changes in hand hygiene compliance rose against the general trend [11]. Similarly, sustained reduction in MRSA rates was also demonstrated by investigators from Singapore where hand hygiene enhancement was implemented as part of a bundle [12].

A systematic review has summarized the literature available until 2009 on the impact of ABHR use on MRSA rates [13]. Among 12 studies included in the review, an increase in ABHR use correlated significantly with an improvement in the MRSA situation (r = 0.78) and was associated with a significant reduction of MRSA rates, whereas no significant correlation was observed between compliance level and MRSA. This latter observation was confirmed by a prospective, observational, ecological study from Ontario, Canada, which also failed to demonstrate a positive ecological impact of improved hand hygiene compliance rates on the incidence of MRSA bacteremia, despite significant improvements in rates of compliance among healthcare personnel [14]. The authors argued that this might be due to both the already extremely low rate of MRSA bacteremia in Ontario at the start of the study and/or the relatively high rates of hand hygiene compliance.

One of the most convincing evidence for the role that hand hygiene compliance plays in MRSA control came from a recent hybrid study involving a prospective interventional cohort study and a randomized controlled trial by Derde and colleagues [15]. They investigated baseline MRSA rates (Phase 1) against the combined effect of enhanced hand hygiene and universal decolonization (Phase 2), as well as the additional impact of screening and contact precautions (Phase 3) on MRSA, vancomycin-resistant Enterococci (VRE) and highly resistant Enterobacteriaceaes (HRE) in 13 European ICUs. Multidrug-resistant organisms (MDRO) rates reduced significantly in phase 2 but did not decrease further in phase 3 with introduction of screening and isolation. Even though the independent effect of hand hygiene enhancement was indeterminable, this was the first cluster-randomized trial to confirm the positive role of hand hygiene enhancement in MRSA control.

### The unknown

Firstly, the delay between improved hand hygiene compliance, increased ABHR use and a subsequent decrease in MRSA cross-infection rates has not been well established and remains open to debate. In an interventional time series analysis, Vernaz and colleagued demonstrated an almost immediate effect of increased ABHR use on MRSA rates with lag times between 0 and 4 months [16]. However the above mentioned study by Johnson and colleagues took more than 2 years of sustained improvement in hand hygiene compliance rate before a favourable effect was seen on MRSA infection rates [5]. Delayed effect by more than two years was also noticed by investigators from CleanYourHands campaign [10]. They proposed two plausible explanations, that this delay might be due to a possible non-linear association between hand hygiene and MRSA prevalence, or due to long term changes in community reservoir of MRSA carriage resulting from the intervention. Moreover hand hygiene campaigns involve education and behaviour change and are therefore unlikely to have a short term effect on MRSA rates.

Secondly, the effect of promoting ABHRs on postoperative surgical site infection due to MRSA might be less significant than previously estimated. In a recent multicentre controlled trial in Europe comparing enhanced hand hygiene with universal MRSA screening, contact precautions and targeted decolonization, hand hygiene promotion on surgical wards outside of the operating theatre did not effectively reduce MRSA rates on its own [17]. However, cessation of this intervention was associated with an increase in MRSA rates suggesting that discontinuing activities to optimise hand hygiene practices may be detrimental. Similarly, in the study from the New Hampshire teaching hospital described above, [11] hospital wide hand hygiene enhancement program did not reduce MRSA surgical site infections attributable to operating rooms.

Thirdly, the incremental benefit of hand hygiene on MRSA after a certain threshold has been reached is unclear. The general assumption of greater hand hygiene compliance yielding greater benefit is being challenged. Cooper and colleagues demonstrated that while a large reduction in ward-level prevalence and colonized patient-days of *S. aureus* is observed when the hand hygiene compliance increases from zero to 20%, minimal additional difference is noticed when the compliance increases above 40% [18]. Another modelling study of transmission of MRSA in ICUs did find that hand hygiene enhancement was the most effective way of reducing MRSA transmission [19]. While this study predicted that the attack rate would increase dramatically if the hand hygiene compliance fell below 40%, similar to the Cooper study they found little benefit with increasing hand hygiene compliance above 48%. The law of diminishing return in improving hand hygiene compliance was also supported by other studies [14, 20]. This finding, however, was in contrast to the Geneva multimodal intervention by Pittet and colleagues [3] which saw a significant reduction in MRSA bacteremia and MRSA clinical cultures with the increase in hand hygiene compliance from 48% to 66%. It must be stressed here that in facilities with low hand hygiene compliance or very high MRSA rates, a campaign promoting ABHR use may still be highly effective.

Lastly, it remains unclear whether contact precautions can be stopped in settings with relatively low MRSA prevalence and sufficient hand hygiene compliance [2]. Good hand hygiene practices may suffer as a result of misuse of gloves and may subsequently increase MRSA rates. Since microbial contamination of healthcare workers’ hands can occur despite the use of barrier gloves, regardless of presence of leaks, hand hygiene remains an important component of appropriate glove use [21–23]. Moreover, recent high-quality studies have questioned the value of patient isolation and contact precautions for effective MRSA control in high endemicity settings [24]. Thus some experts suggest that low MRSA rates can be sustained by promoting standard precautions and good hand hygiene practices only [2]. In contrast, places with a strict ‘search and destroy’ strategy, like the Netherlands, Denmark and Western Australia [25], limit the likelihood of undetected MRSA carriers in hospitals through early preemptive isolation of high risk patients. This renders low hand hygiene compliance rates of limited concern as far as MRSA transmissions from undetected carriers are concerned. However, other pathogens may of course slip through these targeted MRSA early detection and prevention nets, as evidenced by the recent large OXA-48 outbreak in the Netherlands [26]. As such, the hypothesis of whether adequate hand hygiene compliance alone without contact precautions is sufficient to control MRSA transmissions, needs to be tested in large clinical trials in which standard precautions and hand hygiene are tested alone, not as a part of a multimodal intervention as is often the case [15].

## Conclusions

Appropriate hand hygiene during patient care is the primary means of reducing the spread of MRSA. However, further research is necessary to determine the quantitative association between increased hand hygiene compliance, ABHR use and MRSA reduction as well as the role of improving hand hygiene only, independent of contact precautions, for MRSA control.
